# Derivation of harmonised high-level safety requirements for self-driving cars using railway experience

**DOI:** 10.1038/s41598-022-26764-0

**Published:** 2022-12-26

**Authors:** Aleš Filip, Roberto Capua, Alessandro Neri, Francesco Rispoli

**Affiliations:** 1 University of Pardubice, Studentská 95, 53210 Pardubice, Czech Republic; 2 Sogei S.P.A., Via Mario Carucci N. 99, 00143 Rome, Italy; 3 Radiolabs, Corso d’Italia 19, 00198 Rome, Italy

**Keywords:** Engineering, Electrical and electronic engineering

## Abstract

The development and manufacture of self-driving cars (SDCs) have triggered unprecedented challenges among car manufacturers and smart road operators to accelerate awareness and implementation of innovative technologies for cooperative, connected and automated mobility (CCAM), especially those with a high level of automation and safety. Safety improvement is a pre-requisite to justify and unleashing a mass deployment of connected and driverless cars to reach the goal of zero-accident in 2050 set by the European Commission. Behind these motivations a well-justified and widely acceptable high-level safety target for SDCs is mandatory. The aim of this article is to contribute to the derivation of an harmonised high-level safety target for SDCs, starting from the safety requirements and the state of the art achieved by train and airplane operations. The novelty of our approach is to leverage the Common Safety Method-Design Targets (CSM-DT) already adopted and widely accepted by the railway community. According to this approach, the derived, justified and harmonised high-level design safety target for SDCs, defined as the average probability of a dangerous failure PF_SDC_ per 1 h, should be 1 × 10^−7^/h. An example of PF_SDC_ allocation to individual SDC safety functions, including position determination based on Global Navigation Satellite System (GNSS), is described using a fault tree. The proposed methodology can speed up the validation and certification process needed to authorise the SDCs, by capitalising the know-how and best practices in use since many years for the train management.

## Introduction

Advanced driver assistance systems (ADAS) are being extensively installed by car manufacturers in their new models. While ADAS with a lower level of driving automation (Level 1 or Level 2) according to SAE (Society of Automotive Engineers) mainly increases driver comfort and safety during certain tasks, such as parking or lane keeping, higher automation levels starting from Level 3 leading to fully self-driving cars (SDCs) are more demanding in terms of safety with the introduction of automatically commanded steering functions (ACSFs). It is generally assumed that SDCs should be as safe as traveling by train or plane^1^, which can be characterized by a safety risk of 30.0 passenger deaths per billion hours on a train or 30.8 passenger deaths per billion hours on a plane^2,3^. For clarity, these are the risks of train or air travel in the UK for the period 1998–99, when railways were perceived safe (unlike other years in the period 1990–2000). Despite recent advances in aviation and rail safety^4^, a risk of 3 × 10^−8^ deaths/ h has been assumed the target for the SDC acceptable safety level resulting into a major challenge for the automotive industry.

However, to improving the safety a cost must be taken into account for designing and implementing functions satisfying the high safety levels with a technically feasible architecture that must be validated and certified. This task is even more complicated in the case of SDCs. First, millions and millions of different driving situations can be expected that manufacturers are unable to properly test in a reasonable time to provide convincing evidence of meeting the required SDC safety. Second, environmental conditions can be very harsh—such as snow and ice on roads, low visibility due to fog, glare of optical sensors in the setting sun, electromagnetic interference, etc. Third, there is strong pressure on the car’s conformity assessment process, which usually takes no longer than 1 year^5^ and almost the same time is expected for introducing SDCs with ACSFs. The scenario is different in aviation, where it can take more than 5 years to demonstrate compliance with the required regulations and standards for large civil aircrafts. And it also takes years on the railways to prove safety and to certify complex safety-related systems.

The development and implementation of any safety system basically consists of three main phases: (1) specification of system safety requirements, (2) system design and manufacture, and (3) demonstration and approval of system safety. Proper and well defined specifications of the safety requirements at system level are a crucial task as it can significantly affect the overall design of the system and its approval impacting on the final safety and cost of the system. If the safety requirements are underestimated, the system will not be as safe as required, with the consequences of fatal events. On the other hand, if the safety requirements are too conservative, the cost of the system will be excessive penalising its competitivity.

A system is considered safe if the risk associated with the system is acceptable. At present, there are no standard risk acceptance criteria (RAC) in the automotive sector to be taken as reference to specify system safety requirements and to assess the safety of driverless cars. There is also a lack of consensus among manufacturers of automated driving systems on the target system safety requirements (design targets) for comparing different automated driving systems built by different vendors or by public authorities for regulatory purpose.

To overcome these limitations, we have analysed the current practices adopted by other transport sectors characterized by up-front high safety integrity and dependability requirements fulfilling the certification rules. In particular in the railways sector (or aviation) for inspiration to derive a harmonised safety target for SDC being high-level safety requirements clearly specified, well justified and harmonised.

The functional safety of technical systems is generally ensured by a safety management process according to relevant functional safety standards—e.g. IEC 61508^6^ applicable to all industries, EN 5012x^7–10^ used on railways, ISO 26262^11^ used in the automotive industry, etc. In addition, e.g. on a railway, in the case of a significant change in the safety-related signalling system, a risk management process in accordance with the Common Safety Method for Risk Assessment and Risk Assessment (CSM-RA)^12^ and possibly other methods such as the Common Safety Method—Design Targets (CSM-DT) ^13,14^ shall be applied. The great advantage of using CSM-RA and CSM-DT is that they allow the specification and demonstration of harmonised safety requirements, which contributes to ensuring the required interoperability on European railways. Since widely acceptable safety targets for SDCs are still missing, the aim of this article is to describe a new approach to derive harmonised high-level safety requirements for SDCs based on the risk acceptability criteria used in rail and civil aviation.

The practical applicability of the proposed methodology is demonstrated by two examples. The first example shows the derivation of a harmonized design safety target for SDC based on the use of CSM-DT and car accident statistics, and the second example describes the allocation of the safety target to the basic automated driving functions. Assumptions and parameters for estimating the failure probabilities of the basic automated driving functions are given.

The article is arranged as follows: the second section summarises the basic requirements of society for SDC safety. Harmonisation of acceptable risk and safety requirements on European railways is shown in the third section. It includes an overview of risk acceptance principles and criteria, techniques for risk harmonisation of railway technical systems within the CSM-RA, the CSM-DT as harmonised risk acceptance criteria, and classification of catastrophic and hazardous/ critical failures. In the fourth section, a new methodology for specifying safety requirements for self-driving cars based on the CSM-DT is proposed. The fifth section describes derivation of harmonised high-level safety requirements for SDCs. An example of the average failure probability allocation to the main SDC subsystems, including the vehicle positioning function, is also given. Conclusions are formulated in the sixth section.

## Socially acceptable safety of self-driving cars

A risk acceptance criterion, which is a measure of the required safety, is a critical attribute reflecting the willingness of certain users to rely on SDCs. It allows to estimate whether and when driverless vehicles will be mass produced and put into service. How safe should driverless vehicles be for society to accept them? Respondents to a recent survey^1^ expect that driverless cars should be four to five times safer than human driven vehicles. It also implies that the responders expect the global road traffic fatality rate (TFR) should be reduced by approximately 2 orders. The available TFR for year 2018 claims 18.3 fatalities per 100,000 population and year (~ 10,000 h)^15^. It shows that the responders also assume that the acceptable risk associated with a driverless car should roughly correspond to the safety level currently guaranteed by public transport, such as rail or civil aviation.

The safety risk of future automated car driving systems, which consist of a vehicle on-board unit with automated steering, braking and accelerator functions, cooperating with a way-side infrastructure, must be properly measured, controlled, and evaluated. While society recognizes data on mortality caused by existing cars without or with limited grade of automated steering, it is likely that there will be almost zero tolerance for any fatal accidents due to possible technical failure of the ACSF. If the above global value of TFR should be reduced by 2 orders and expressed per 1 h, then it corresponds to TFR_reduced_ = 0.18 × 10^−9^/ h. One of risk acceptance criteria that is called MEM (Minimum Endogenous Mortality) and which has been widely used to assess railway safety, especially in Germany, assumes that no single technical system should contribute more than 1 × 10^−5^ fatality/ year, i.e. approximately 1 × 10^−9^/ h^8^. In some cases a magnitude of the target individual risk (TIR) of fatality used to determine the railway tolerable hazard rate (THR) is conservatively set less than 1 × 10^−9^/ h—e.g. 1 × 10^−10^/ h^16,17^. Sometimes this is also justified by the assumption that 10% of the total risk (1 × 10^−9^/ h) is allocated to railway signalling, or an additional safety factor of 10 is added to the TIR^17^. It independently confirms the fact presented in the first section that the socially acceptable safety level of future driverless cars estimated in^1^ should be approximately at the same (high) level as is common on railway.

## Harmonisation of risks and safety requirements

### Railway experience

Railways belong to a regulated and very safe transport sector^18^ and since the very beginning, railway safety has been based on conservative principles and worst-case approach. The worst-case approach takes into account many scenarios and assumptions that are unlikely to occur simultaneously. Railway technical systems shall be sufficiently safe, but they must be not safer than is actually required, otherwise they would be more expensive, and no one would use them. In addition to safety, great attention is also paid to the efficiency of rail transport.

The European Railway Traffic Management System (ERTMS) is a centralised command and control system conceived to prevent human errors and was designed more than 25 years ago to manage railway operations safely and efficiently across borders between different European countries having their own proprietary system. The ERTMS system authorizes the train to move to a predetermined point as soon as the train position is determined, and all the safety conditions are met. The position of train compliant with safety integrity level (SIL) 4 and THR < 1 × 10^−9^/ h is determined by an on-board odometer, the errors of which are periodically reset by means of transponders (balises) installed along the railway.

A key feature of the ERTMS is to ensure interoperability^19^ among on-board and track-side subsystems shared between different actors, in particular infrastructure managers and railway undertakings. The same requirement is applicable for ensuring interoperability between car systems and road infrastructures. The high safety and dependability requirements for ERTMS must be met—also in cases where track balises are replaced by virtual balises and detected by GNSS-based positioning. It is necessary to go through a certification and authorization process that guarantees compliance with all ERTMS requirements—i.e. CENELEC (Comité Européen de Normalisation Électrotechnique) railway safety standards, technical specifications for interoperability (TSIs), EU regulations, directives, etc.). A clear specification of the system safety requirements is therefore essential.

Most importantly, European railways already use the concept of Common Safety Targets (CSTs)^18^, which in fact means the minimum levels of safety that the railway system should achieve. CSTs are therefore more general and do not only concern the technical system. In addition, railways are also recommended to use the so called Common Safety Method Design Targets (CSM-DT)^13,14^ which are actually harmonised semi-quantitative safety requirements for railway safety systems when explicit risk estimation is performed—i.e. when there is insufficient experience with the new system. CSM-DT are consistent not only with the current European safety levels used in the quantitative assessment of railway risks, but also with the design targets used in aviation. As will be shown below, the use of CSM-DT in the automotive industry can also help significantly simplify the derivation of high-level safety requirements for SDC.

### Risk acceptance principles and criteria

**Figure 1:**
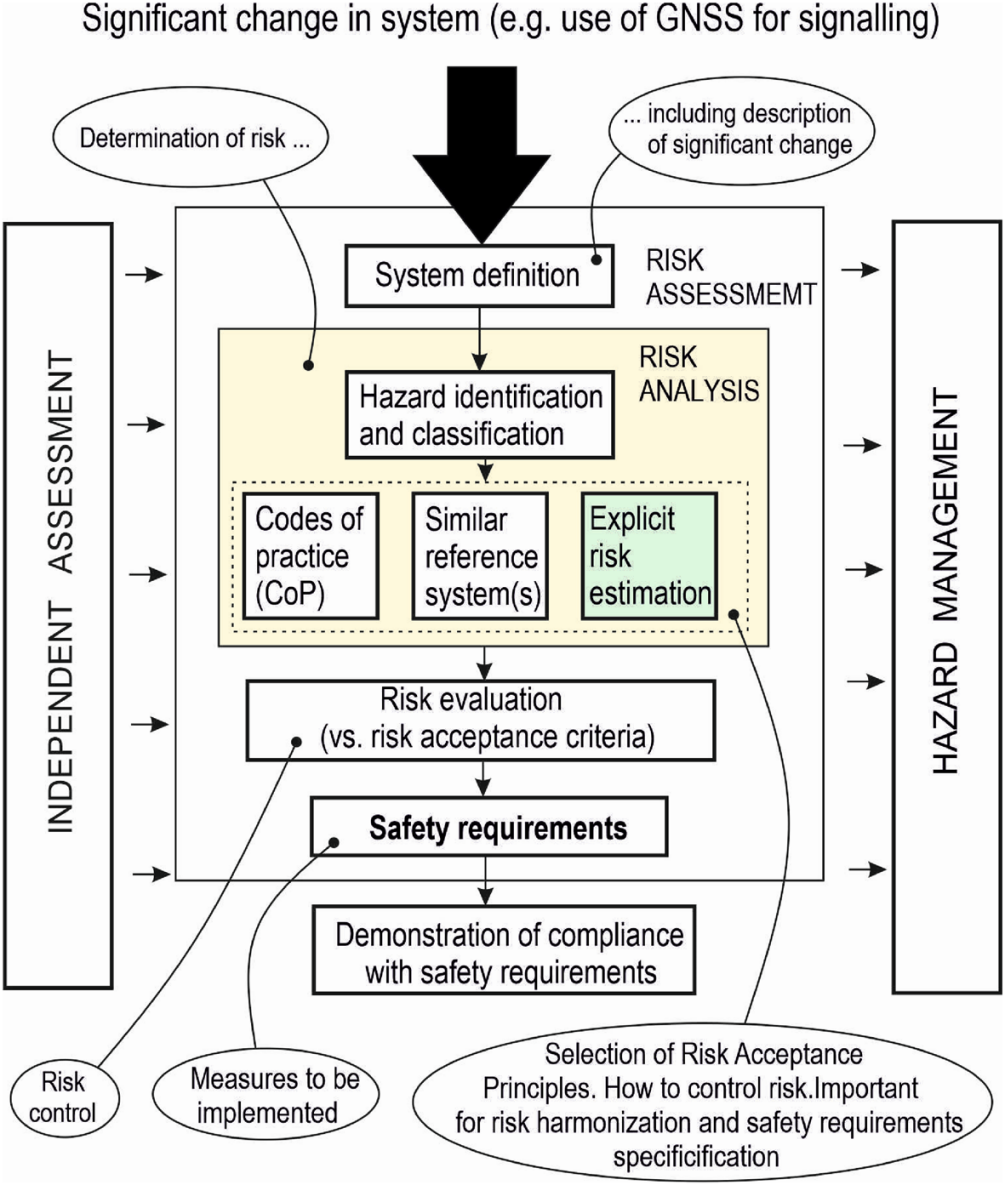
Diagram of common safety method for risk evaluation and assessment.

Railway stakeholders must safely manage all changes to upgrade the ERTMS using the so-called Common Safety Method for Risk evaluation and Assessment (CSM-RA)^12^ according to European railway regulations. This also applies to the above-mentioned integration of GNSS with ERTMS for virtual balise detection. A diagram of CSM-RA is shown in Fig. 1. The main part of the CSM-RA is the risk assessment process, the output of which is the harmonised safety requirements for the system. The risk assessment is a responsibility of the system change proposer, e.g. railway infrastructure manager or equipment manufacturer/supplier. Hazard management is provided in operation by the railway infrastructure manager or train operator using a safety management system. Risk assessment and hazard management form the risk management process. An independent assessment body (AsBo) supervises the correct application of CSM-RA.

CSM-RA harmonises the risk management process across Europe and leads to harmonised safety requirements for safety systems. It differs from the safety management process that must be performed for the railway safety-related system according to the railway CENELEC safety standards EN 5012x^7–10^. CSM-RA is applied at the level of the whole railway system, whereas the safety management process concerns safety-relevant systems. Nevertheless, the CSM-RA complies with the CENELEC EN 5012x. Risk harmonisation is ensured through the following three risk acceptance principles (RAP) and risk acceptance criteria (RAC): codes of practice (CoP), similar reference systems and explicit risk estimation (Fig. 1).

In addition to railway systems, harmonisation of risks and related technical safety requirements is also important in the field of self-driving cars, as it leads to the specification of widely acceptable safety requirements. Consensus on the car safety requirements is a pre-requisite to promote technical interoperability and also to facilitate the type-approval process in this field.

### Explicit risk estimation

Harmonisation of risk acceptance and specification of safety requirements in land transport, such as rail or road, is crucial for the system for complying with the safety requirements and also for achieving the required efficiency. Compared to risk assessment of driverless cars, railways have undergone a process of harmonisation of risk acceptance over the last few decades and have also developed a basic framework for a safety certification and approval process for advanced technical systems (TS). As mentioned above, the agreed RAP and RAC are the main means of harmonising and mutual recognition of safety requirements^12^.

Widely acceptable CoP, such as ERTMS Technical Specifications for Interoperability (TSIs), CENELEC safety standards, etc., used as RAP, make it possible to harmonise risk and thus railway safety requirements across Europe—widely accepted world-wide (see Fig. 1). These CoPs have been developed on the basis of the experience of designing and deployment of ERTMS systems on about 110,000 km of lines, 50% of which are outside of Europe.

Reference systems can be used as RAP in a very similar way as CoP because a reference system is a system that has demonstrated an acceptable level of safety in practice. Both CoP and similar reference systems used as RAP can also be considered as risk acceptance criteria (RAC).

**Figure 2:**
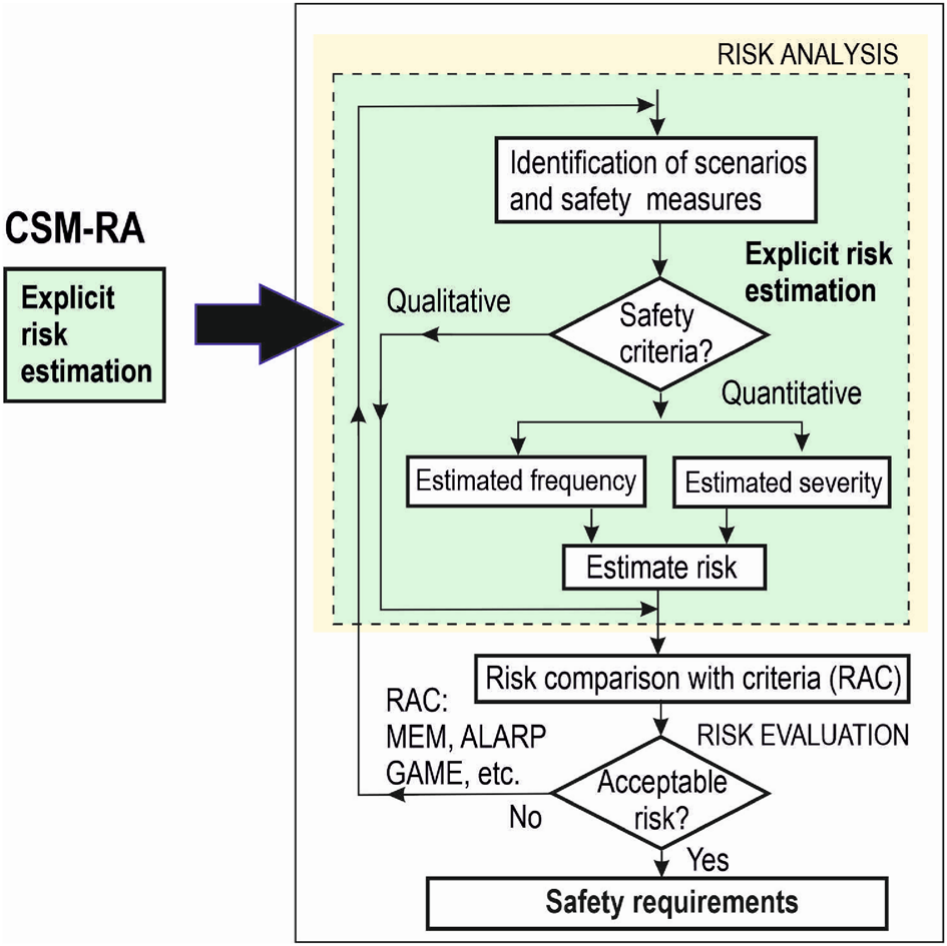
Flowchart of explicit risk estimation.

In the absence of proven return of experience in the design and evaluation of a specific safety system, as is the case of SDCs, an explicit risk estimation should be used as a RAP. A flowchart of explicit risk estimation is shown in Fig. 2. Risk is explicitly estimated either qualitatively, especially in the initial phase of risk estimation when there is not yet sufficient data on the system, or quantitatively by estimating the frequency of hazardous events and their severity. To determine the safety requirements for the system, specific railway RAC are then needed—e.g. MEM, ALARP (As Low As Reasonably Practicable), GAME (Globalement Au Moins Équivalent), etc.^8^. The problem is that these RAC are not harmonised in Europe. Therefore, the associated risk with a given safety system may not be acceptable in all EU countries. This means that it is also not possible to harmonise the resulting safety requirements for TS. As outlined in the following section, widely acceptable RAC are needed.

### Design safety targets for technical systems

In the rail domain, it was obviously necessary to ensure mutual recognition of risk assessment of technical systems (TS) when explicit risk estimation as RAP is used. In order to harmonise safety requirements for the design of E/E/PE (Electric/ Electronic/ Programmable Electronic) safety-related systems, CSM Design Targets (CSM-DT)^13,14^ have been introduced by the EU Agency for Railways (ERA). The CSM Design Targets are harmonised RAC for TS. The term ‘Design Targets’ was introduced to distinguish the acceptance of risks associated with technical systems from the acceptance of operational risks and the overall risk at the railway system level.

CSM Designed Targets are defined in terms of the frequency of dangerous failure (FF) of TS as shown in Table 1^13^. There are used by system designers and manufactures to answer the question: ‘Is my system safe enough?’. The goal of the harmonised CSM-DT is to assure that the designed TS is safe enough, as it is required by society. At the same time, the TS will not be safer than actually required.

**Table 1:** Common safety method—design targets for railway technical systems.

Number of people affected by accident (exposed to risk)	Large number of people	Very small number of people
Multiple fatalities	Class (a) **FF = 1 × 10**^**−9**^**/ h** (catastrophic accident)	Class (b) **1 × 10**^**−7**^**/ h** (critical accident)
At least one fatality	Class (a) **FF = 1 × 10**^**−9**^**/ h** Class (b) **FF = 1 × 10**^**−7**^**/ h** (low train speed, low traffic, …)	Class (b) **1 × 10**^**−7**^**/ h**

Significant values are in [bold].

The CSM-DT were derived on the basis of current experience and best practice in the design of railway safety systems and are only applicable to functional failures that directly lead to accidents. CSM Design Targets represent harmonised functional safety requirements for TS and apply to both random failures and systematic failures^13^. Design targets are used as semi-quantitative safety requirements for random HW failures of E/E/EP technical systems. The associated systematic failures shall be managed by safety and quality processes in accordance with the required safety integrity level (SIL) corresponding to the design target. The relationship between FF and SIL is defined e.g., by the SIL table in IEC 61508 or EN 50129. A similar table for automotive SIL (ASIL) is in ISO 26262. It is therefore clear that CSM-Design Targets can also be applied to systematic failures due to software errors, which are a major problem in modern safety systems.

According to Table 1, there are two classes of failure frequencies: Class (a) with FF = 1 × 10^−9^/ h and Class (b) with FF = 1 × 10^−7^/ h. The relevant FF class is determined by the estimated risk associated with the technical system, i.e. by the number of persons exposed to the hazardous event and the expected number of deaths.

It should be noted that the failure frequency (FF) corresponds to the rate of occurrence of failures (ROCOF). It is also called unconditional failure rate/ intensity of an item at time t and is often denoted by w(t)^6^. ROCOF also means the expected (mean) number of failures of an item over a time interval dt and can be written as E[N(t + dt)−N(t)]/dt, where N(t) is a number of failures in time t. Since the design safety targets for SDCs only apply to hazardous failures that directly lead to an accident, then the FF will correspond to the probability of a hazardous failure (PF) for a given time interval, e.g. 1 h.

Furthermore, the hazard rate (HR), which is also used as a safety attribute for safety-related systems, is the frequency of a dangerous failure, provided that no failure has occurred until time t. In fact, it is the frequency of failure conditioned by the reliability of the system. Automated car driving systems are generally considered to be systems with high reliability practically equal to 1, and therefore FF, PF and HR can be further considered identical.

The automotive functional safety standard ISO 26262^11^ defines the probabilistic metric for random hardware failures (PMHF) as a measure of safety for automotive items. The PMHF corresponds to the average probability of failure per 1 h over the operational lifetime of the item. Therefore, the harmonised high-level safety target for SDCs is expressed as the average probability of failure per 1 h. For completeness, it can be added that ISO 26262-10 shows that the automotive PMHF corresponds to the failure rate (or HR in case of dangerous failures), which is used, among others, as a safety attribute for railway TS. There is nothing to prevent that the railway high-level CSM-DT defined by FF can also be used as design safety targets for self-driving cars. As mentioned above, FF is more concerned with estimating the high-level risks associated with the use of TS, whereas HR or PF (PMHF) are used to specify the safety parameters of TS. PF is further used as a safety target for self-driving cars.

Therefore, the question is how to use harmonised design targets. First, hazard rate of a specific functional hazardous failure of a technical system has to be estimated. For example, failure mode, effects and criticality analysis (FMECA) or fault tree analysis (FTA) are commonly accepted procedures. The estimated HR is then compared with the required CSM Design Target, i.e. FF. If the TS does not comply with the CSM Design Target, changes need to be made to the design of the safety system.

**Figure 3:**
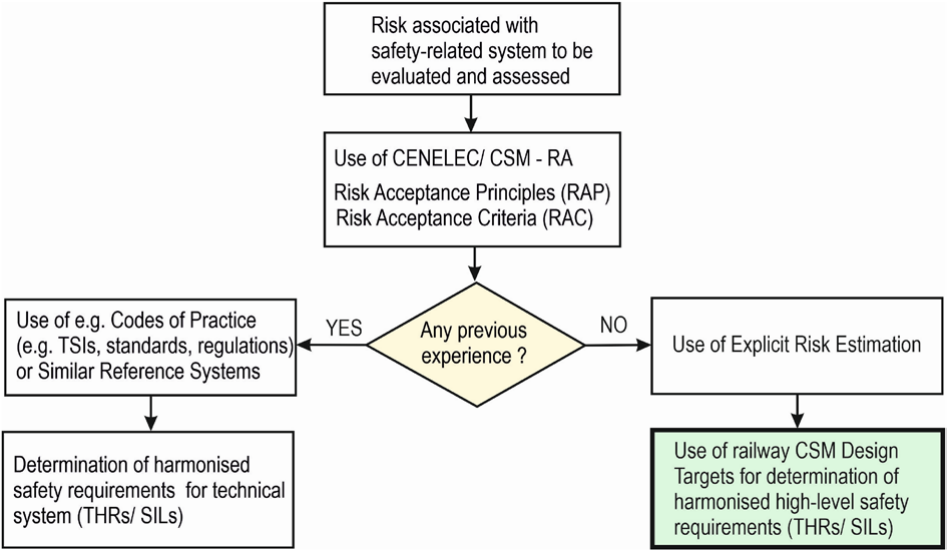
Flowchart for determination of harmonised high-level safety requirements.

For example, in the case of ERTMS with virtual balises (VBs) detected by means of GNSS, harmonised risk acceptance principles (RAP) and harmonised risk acceptance criteria (RAC) in the form of relevant ERTMS TSIs can be utilised to specify safety requirements for VB detection. If a harmonised RAP is not available (e.g. CoP or similar reference system), it is recommended to use harmonised CSM Design Targets for requirements determination as shown in a flowchart in Fig. 3.

A long-term experience with the design of E/E/EP systems with high-safety integrity in the field of automated car driving compared to rail signalling is still lacking. There are neither harmonised RAP nor harmonised RAC that could be used for self-driving cars. For this reason, the use of railway CSM Design Targets in terms of FF is proposed to specify harmonised high-level safety requirements for self-driving cars. In practice, the derivation of a safety target for SDCs described later in this article can serve as a contribution to the expert discussion to specify a uniform safety target for SDCs at the international level. For example, similar to the high-level safety target for ERTMS that has been specified, which is 2 × 10^−9^ dangerous failures per 1 h and per train^20^.

It is essential for the harmonisation of SDC requirements that the CSM Design Targets are in line with the classification of failures agreed in the aviation case. This increases the weight of the harmonisation used for SDCs. A comparison of the classification of failures of technical systems used in rail and aviation is described in the following section.

### Classification of dangerous failures on railway and in aviation

The derivation of aviation and rail system safety requirements usually takes into account the following consequences of failure and the associated failure occurrence:

Aviation:

- Catastrophic failure consequences resulting in multiple fatalities usually with loss of plane (thus impacting all occupants), should not exceed an occurrence of 1 × 10^−9^/ flight hour. Failure consequences are extremely improbable in this case.
- Hazardous failure consequences reducing capability of airplane, large reduction in safety margins, physical distress or excessive workload on crew and impacting a relatively small number of occupants, should not exceed an occurrence of 1 × 10^−7^/ flight hour. Failure consequences are extremely remote in this case.

Railways:

- Failures of functions having possibility to affect whole train (i.e. all occupants) and resulting in fatalities should not exceed an occurrence of 1 × 10^−9^/ 1 h. Failure consequences are catastrophic in this case.
- Failures of functions having possibility to affect a limited area of train (thus a relatively small number of occupants) and resulting in at least one fatality should not exceed an occurrence of 1 × 10^−7^/ 1 h. Failure consequences are classified as critical in this case.

Catastrophic safety risks are generally controlled with safety-related systems compliant with SIL 4 and critical safety risks by systems compliant with SIL 3. It is evident that failure occurrences and consequences in aviation and on railways are classified in a very similar way.

The use of the semi-quantitative railway CSM Design Targets is proposed to harmonise safety requirements for SDCs derived from the target individual risk of fatality (TIR). This solution is described in more detail below.

## Methodology for deriving safety requirements

In order to develop a methodology for deriving high-level safety requirements for SDCs using railway experience, it is first necessary to show how safety requirements can be derived according to the IEC 61508^6^ and EN 50129^10^ safety standards that are used in the rail sector. IEC 61508 is a basic functional safety standard applicable to safety-related systems in all industries that incorporate E/E/PE devices. It is also the parent standard that has been used to create application-specific safety standards such as EN 5012x  for railways, ISO 26262 for automobiles, IEC 61511 for a process industry, etc.

The fundamental safety concept according to IEC 61508 is that any safety-related system must work properly or fail in a predictable (safe) way. The proper functioning of a safety system is based on the correct implementation of safety functions that protect against certain hazards. IEC 61508 specifically covers hazards that occur when safety functions fail. The main objective of IEC 61508 is therefore to reduce the risk associated with a hazardous failure to an acceptable level. IEC 61508 is built on two basic principles: i) the safety lifecycle intended to reduce or eliminate failures due to systematic causes during system development and operation, and ii) the probabilistic failure approach to address dangerous random hardware failures via safety integrity level (SIL).

IEC 61508 assumes that the safety system consists of an equipment under control (EUC) and an EUC safety-related system. Safety functions with continuous or high demand modes of operations are considered, as there are used both in railway signalling and control system of self-driving cars, where the EUC is tightly integrated to the EUC safety-related system. The corresponding safety requirements for safety-related functions in terms of the average frequency of dangerous failure per 1 h (PFH) can be determined by means of the hazard analysis and risk assessment (HARA) from the socially acceptable target individual risk of fatality (TIR). The determined PFH value with respect to the identified hazard for the given operational scenario is then assigned the appropriate SIL, which is a qualitative measure of safety integrity.

The railway standard EN 50129 focuses on the railway safety-related system. Because EN 50129 is a modification of IEC 61508 for railway applications, the quantitative procedure used to derive THR according to EN 50129 is practically the same as the procedure used to determine PFH according to IEC 61508. In the initial phase of HARA, when there is not enough information about the system to be designed, a qualitative approach to the specification of system safety requirements can be used. Later, when more information about the system is available, THR can be calculated e.g. using (1) for individual risk of fatality (IRF) as follows^21^

1 $$IRF_{i} = \mathop \sum \limits_{{all \;hazards H_{j} }} N_{i} \left[ {THR_{j} \cdot \left( {D_{j} + E_{ij} } \right) \cdot \mathop \sum \limits_{{all \;accidents\; A_{k} }} C_{jk} \cdot F_{ik} } \right] \le TIR$$

where IRF_i_ is the individual risk of fatality (per time) for the i-th particular user of the system, N_i_ is the individual usage profile (number of usages per time), THR_j_ is the tolerable hazard rate of the safety function protecting against the j-th hazard H_j_ , D_j_ is the duration of the hazardous system failure and E_ij_ is the exposure time for the i-th individual user and j-th hazard. C_jk_ is the probability of the k-th accident A_k_ caused by the user (e.g. a car driver at a railway level crossing) and F_ik_ is the probability of user’s fatality for the k-th accident. The sum ƩCjk*Fik represents the probability of fatality of a system user during a potential accident when the technical parameters of the system (e.g. duration of hazardous failure or exposure time) are not yet applied.

In many cases, the above general approach to quantitative risk analysis to determine THR / SIL can be replaced by widely acceptable and harmonised risk acceptance principles such as codes of practice (e.g. ERTMS TSIs), similar reference systems, or the harmonised CSM Design Targets. These approaches to the specification of system safety requirements are based on the long-term experience of railways with safety-related systems. This is also the case of GNSS-based ERTMS, where the ERTMS TSIs and the related technical subsets containing safety requirements for the baseline ERTMS have been used to specify safety requirements for virtual balise detection and GNSS in the H2020 ERSAT GGC project^22^.

To overcome the lack of experience with the safety of self-driving vehicles, the railway CSM-DT is proposed to harmonise the safety target. The application of the CSM Design Targets, which are also fully aligned with aviation safety measures (in terms of failure consequence classification and required failure rate), can only strengthen the process of harmonising risks and requirements. The proposed methodology for deriving and harmonising high-level safety requirements for SDCs consists of the following steps:

- Specification of a socially acceptable level of safety of SDC based on a comparison with the generally accepted level of safety of other means of transport. Answering the question: ‘How safe should driverless cars be for society to accept them?’.
- Using the actual safety performance, expressed as fatality risk, chosen for the safest means of transport, i.e. rail and aviation, to set the safety target for SDC. This is the result of the public opinion survey mentioned above^1^.
- Moving from an acceptable target risk of fatality to an acceptable number of fatal car accidents per 1 h and subsequent average probability of dangerous car failure (PF) per 1 h. This is based on car accident statistics.
- Application of the appropriate risk acceptance principle (RAP) and risk acceptance criteria (RAC) to harmonise the target average probability of failure per 1 h for SDC (PF_SDC_). The CSM Design Targets were selected as the RAP/RAC for this purpose.
- Allocation of the PF_SDC_ value to the probabilities of failure of SDC safety functions, including e.g. GNSS-based positioning.

The next section describes how the railway CSM Design Targets can be practically used to determine and harmonise safety requirements for SDCs.

## Derivation of harmonised safety requirements for SDCs

### High-level design safety target

**Figure 4:**
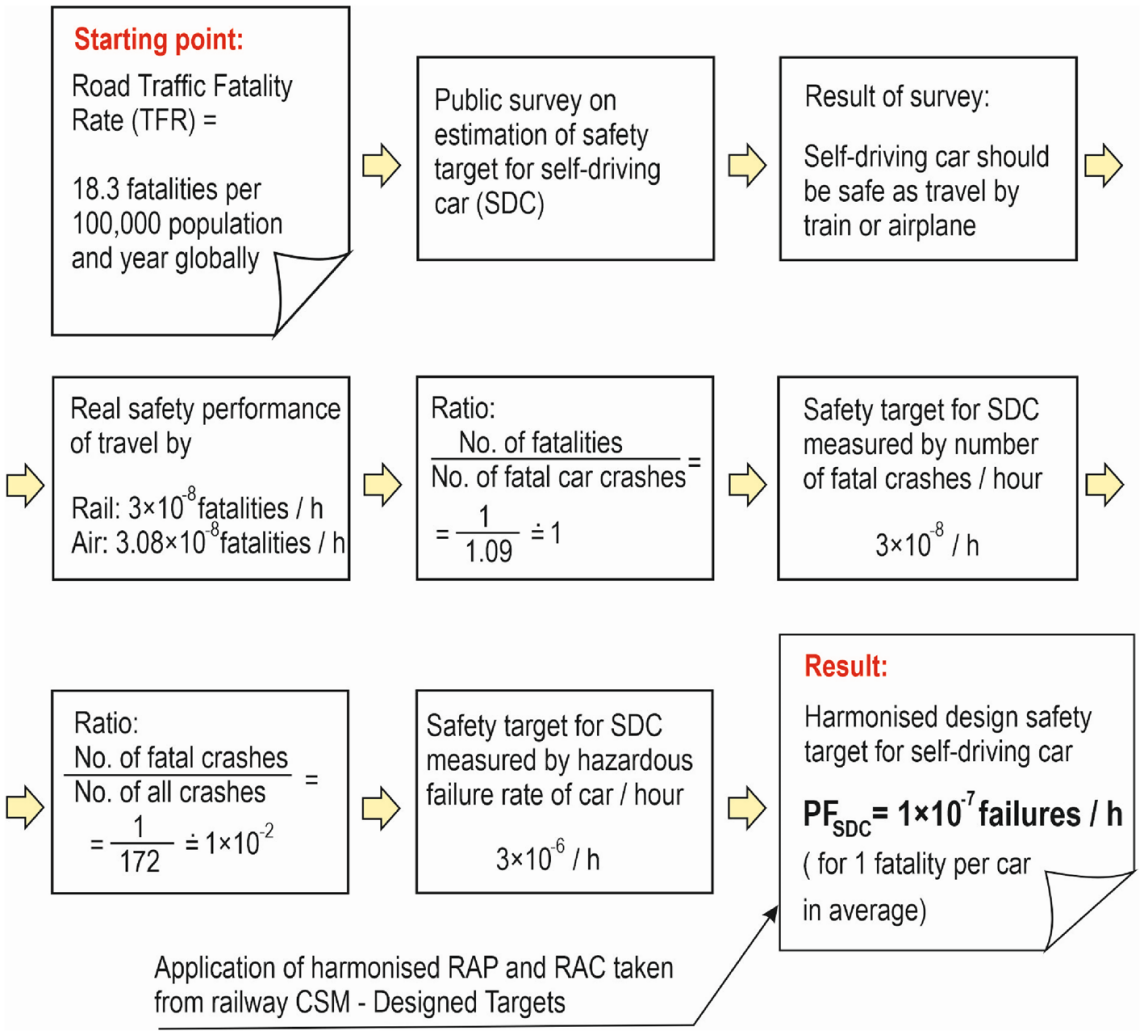
Procedure for derivation of harmonised design safety target for self-driving cars.

The proposed procedure for deriving the high-level design safety target for self-driving cars (SDCs) is outlined in Fig. 4. The aim of this procedure is to get a harmonised average probability of dangerous failure of self-driving car (PF_SDC_), which will be further allocated to the main SDC safety subsystems. The procedure is based on the following three facts: (i) the actual safety performance of road transport as expressed by the world traffic fatality rate (TFR)^15^, (ii) users' expectations on the safety of future SDCs resulting from a recent public survey^1^, and (iii) the harmonisation of systems safety requirements using the Common Safety Method Design Targets (CSM-DT)^13,14^.

The purpose of using the risk acceptance approach based on harmonised CSM Design Targets is to derive a widely acceptable design safety target for self-driving cars. The advantage of the proposed solution is that the harmonisation of safety requirements for SDCs is carried out in line with the safety levels approved for railway and civil aviation use.

The starting point in this procedure is the world TFR^15^ as a measure of road safety, which serves as a baseline reference for further increasing the safety requirement for SDCs based on the results of the public survey described in^1^. It should be noted that this measure of safety risk is not expressed in kilometres or miles travelled, but in population and year. Subsequently, the conclusions of the public survey on the estimation of the required safety level for self-driving cars are considered. The conclusions state that the level of safety of SDCs should be approximately the same as the level of safety of air or train travel. In this analysis, the safety performance of traveling by rail or plane is expressed in 1 h rather than in the distance travelled. This is because human safety is usually evaluated per time—e.g. by means of RAP/RAC such as MEM. Actual safety performance when traveling by plane or train expressed by the risk of 3 × 10^−8^ fatalities of travellers/ h should be rather considered as a tolerable risk, but not as an acceptable risk. Tolerable means that society can live with it but cannot be considered as negligible or as something that can be ignored. The risk should therefore be further reduced if it is possible, e.g. using the ALARP principle. Acceptable risk means that everyone who may be affected is prepared to accept it, provided that no further changes in risk control mechanisms are required. However, only the term ‘acceptable’ is used in the remainder of this article. This is because the RAC is finally used to harmonize the safety target for SDC. The use of RAC (CSM-DT) in this study also verifies the correctness of the selection of air or train travel risk data, as the harmonised safety targets set for SDCs cannot be worse than those that are socially acceptable.

In railway safety systems, RAP/RAC are usually introduced at the beginning of the requirements derivation process, as can be seen for example from the target individual risk (TIR) used in Eq. (1). The TIR can be specified e.g. using MEM with an acceptable probability of death of 1 × 10^−9^/ h, which applies to the wider population, not just passengers. Then the actual risk of air or rail travel (3 × 10^−8^/ h) is higher than the generally acceptable safety risk, e.g., 1 × 10^−9^ fatalities/(person × h) or less, used for rail system design.

As this requirements specification process starts from the real safety performance of travel by train or air, which results from the findings of the public survey mentioned in the second section^1^, RAP/RAC cannot be applied at the beginning of the process. The CSM-DT approach specifies the design safety targets for a technical system in terms of dangerous failure frequency per 1 h and not in terms of the number of fatalities per 1 h. For this reason, CSM-DT used as RAP/RAC for SDCs is applied at the end of the requirements specification process, as shown in Fig. 4.

And how can the acceptable risk (expressed in number of deaths) as a measure of safety for SDC be converted into a probability of SDC failure? Based on accident statistics, it can be assumed that approximately one fatal car accident causes one death^23^. Then the acceptable safety risk measured by the number of deaths per hour (3 × 10^−8^ deaths/ h) is converted to the probability of a fatal car accident per hour (3 × 10^−8^ fatal car accidents/ h), as shown in Fig. 4. For example, in aviation, not every dangerous failure leads to an accident. This describes the ratio of fatal accidents to incidents. In deriving the aviation integrity risk requirement for GNSS, the ratio is 1:10. Also, in the case of a car, not every critical failure will cause a fatal accident. The ratio of fatal car accidents to total car accidents based on statistical evaluation is 1:172^23^. This ratio is conservatively chosen as 1:100 (Fig. 4). The occurrence of a fatal car accident per 1 h (on average with approximately one fatality) is thus converted to the occurrence of a critical failure per 1 h, which is 3 × 10^−6^ critical failures / h / car. It is now necessary to assess whether this value is also acceptable on the basis of long-term experience in building and operating safety systems.

Due to the lack of experience with safety systems and safety targets for driverless cars, the CSM Design Targets approach is used as the risk acceptance principle (RAP) and risk acceptance criteria (RAC) for SDCs. It is assumed that on average one death occurs during a fatal accident and that the accident affects a small number of people. According to Table 1, the above assumption corresponds to the design target of Class (b) with an average probability of dangerous failure PF_SDC_ of 1 × 10^−7^/ h/ car. This is the harmonised high-level design safety target for the whole SDC. The consequences of a failure are classified as critical in this case.

### Example: Allocation of high-level safety target within SDC

**Figure 5:**
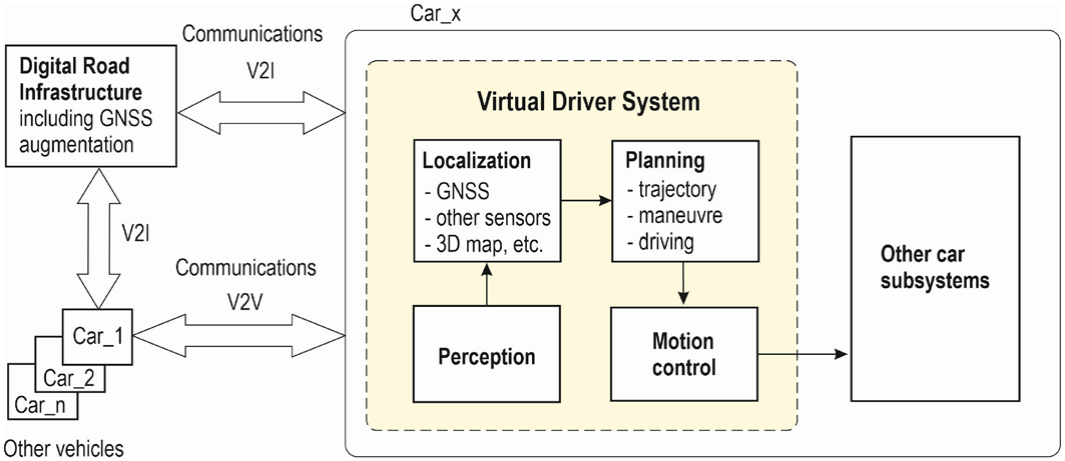
Logical model of automated car driving.

**Figure 6:**
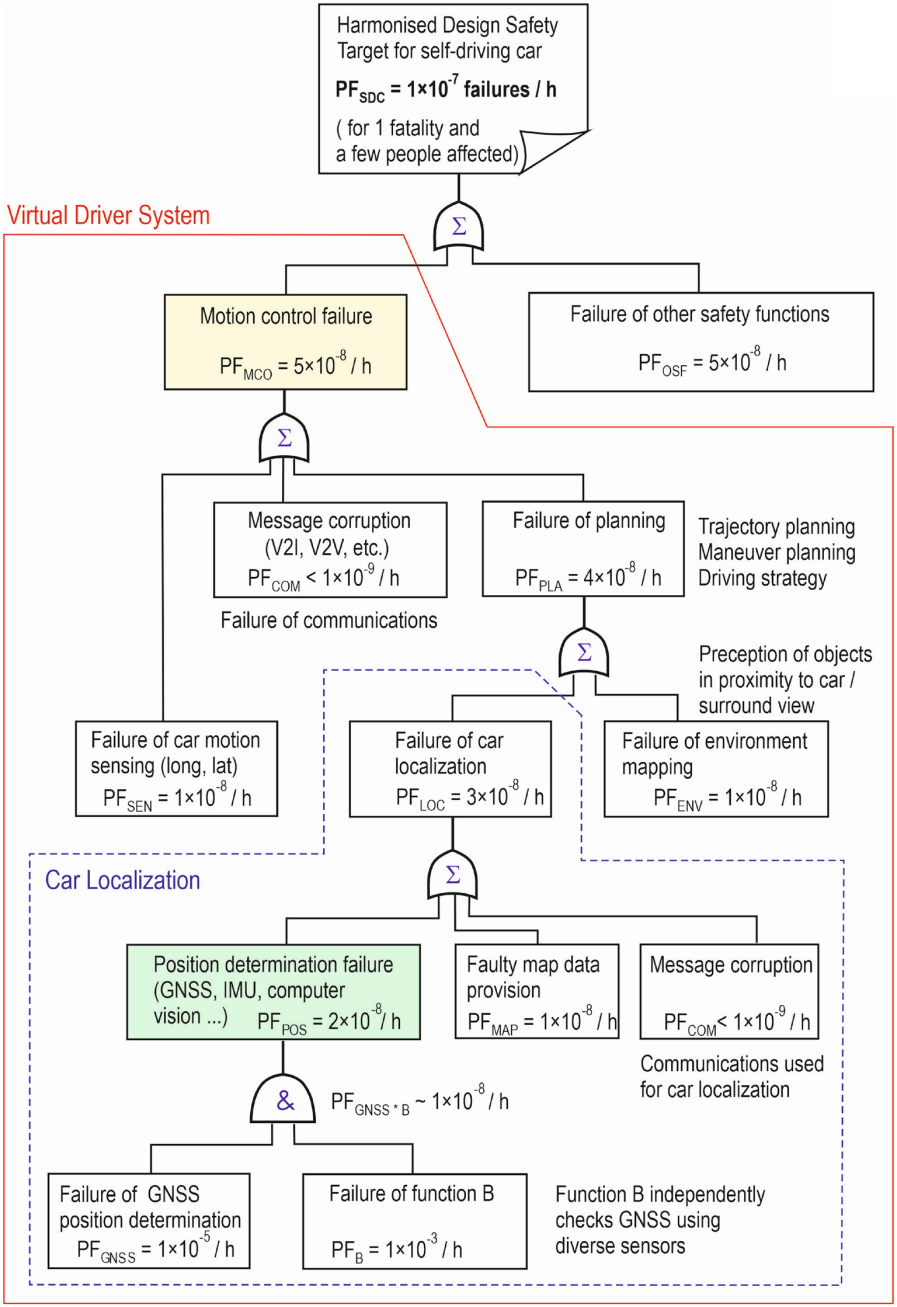
Example of fault tree with allocation of safety target to main SDC functions.

The allocation of a high-level safety target to individual safety functions implemented by SDC subsystems depends on a logical model of automated driving, which usually consists of a virtual driver system and other car subsystems. An example of the logical model is shown in Fig. 5^23^. The communication of the depicted vehicle (Car_x) with the digital road infrastructure and other vehicles (Car_1, Car_2, etc.) is also indicated in Fig. 5, because a possible communication failure has to be considered in the following safety analysis, as e.g. further shown in the fault tree analysis (FTA) in Fig. 6.

In general, the data flow for automated driving can be summarized as follows: the environment and physical situation are observed by various sensors such as cameras, GNSS, IMU (Inertial Measurement Unit), LiDAR (Light Detection And Ranging), etc., and the measured sensor and infrastructure data are then analysed and processed in a software system to determine a safe and dependable feedback to control functions of the driving system. How the software system is composed in detail depends highly on the set of hardware sensors and the required functionally of the system. The virtual driver system (Fig. 5) consists of the following subsystems: perception, car localization based on augmented GNSS, route planning and vehicle motion control. Vehicle-to-infrastructure (V2I) and vehicle-to-vehicle (V2V) communications support the connected car concept including the use of augmented GNSS for high accuracy and safety integrity of the vehicle positioning function.

An example of a fault tree with the allocation of a high-level design safety target for SDC, i.e. PF_SDC_ of 1 × 10^−7^/ h, to main safety functions of SDC is shown in Fig. 6. The allocation of PF_SDC_ has been performed according to the logical model of automated car driving outlined in Fig. 5. PF_SDC_ of 1 × 10^−7^/ h is equally allocated between PF_MCO_, which is the probability of motion control failure, and PF_OSF_, which is the probability of failure of other vehicle safety functions. PF_MCO_ of 5 × 10^−8^/ h is further allocated to PF_SEN_ of 1 × 10^−8^/ h, i.e. the failure probability of car motion sensing, PF_COM_ < 1 × 10^−9^/ h, i.e. the failure probability of message corruption (related to communications V2I, V2V, etc.) and PF_PLA_ of 4 × 10^−8^/ h, i.e. the probability of motion planning failure. PF_PLA_ of 4 × 10^−8^/ h is allocated between PF_LOC_ of 3 × 10^−8^/ h, i.e. the failure probability of car localization function and PF_ENV_ of 1 × 10^−8^/ h, which is the probability of failure of the car environment mapping. PF_LOC_ is further divided among PF_POS_ of 2 × 10^−8^/ h, i.e. the probability of position determination failure, PF_MAP_ of 1 × 10^−8^/ h, i.e. the probability of incorrect map data provision, and PF_COM_ < 1 × 10^−9^/ h, i.e. the probability of message corruption failure related to the communications used to locate the car. Safe car positioning is achieved by a logical AND combination of the GNSS based positioning function with PF_GNSS_ of 1 × 10^−5^/ h and the so-called Function B with PF_B_ of 1 × 10^−3^/ h.

The Function B, which independently detects GNSS failures, is needed because safety integrity of GNSS positioning may be compromised by local effects such as multipath, electromagnetic interference, intentional jamming, etc. This diagnosis allows to meet the demanding requirement for the integrity of car localization (PF_LOC_ of 3 × 10^−8^/ h). It is assumed that the GNSS and B-function hazardous failures are statistically independent due to the different sensors used (GNSS, odometry, vision sensors, etc.). Then the logical AND operator can be used to combine the failure probabilities of GNSS and Function B. In case of GNSS, a conservative estimate of PF_GNSS_ of 1 × 10^−5^/h can be justified, e.g. by converting the aviation GNSS integrity risk requirement for Category- I operations (for GNSS signal and GNSS onboard equipment) of 3.5 × 10^−7^/ 150 s^24^ to 8.4 × 10^−6^/h , which is approximately 1 × 10^−5^/h (PF_GNSS_). The following relation is used for the conversion: 3600 s = 24 × 150 s. Based on railway experience with ERTMS and virtual balise detection using GNSS^25^, which employs safe data communications and high integrity maps, the failure probabilities associated with map data and message corruption are assigned as PF_MAP_ of 1 × 10^−8^/ h and PF_COM_ < 1 × 10^−9^/h. Then a probability of failure of the car localization function of 3 × 10^−8^/ h (PF_LOC_) can be achieved. Failure probabilities related to motion control, motion sensing, planning and other functions are assigned to meet the harmonised design target for SDC (PF_SDC_ of 1 × 10^−7^/ h).

## Conclusions

A key challenge for deploying self-driving cars (SDCs) is to increase the level of safety of traditional cars. However, there is currently no generally acceptable high-level safety target that driverless cars manufacturers must meet in real-world applications. To fill this gap, we proposed to re-use the expertise and procedures developed for the ERTMS railway system, widely deployed world-wide in the last 20 years without major failures, as inspiration to derive a harmonised safety target for SDCs.

The authors propose an approach to derive a widely acceptable high-level safety target for SDC in terms of average probability of hazardous failures per hour, using the railway experience in harmonizing risk and system safety requirements. To do this, public views on the desired safety of the driverless car, statistical data on car accidents and railway common safety methods (CSM-RA and CSM-DT) were used. The derivation follows the findings of a recent public survey, according to which respondents believe that the acceptable risk associated with a driverless car should correspond to the level of safety achieved by public transport, such as rail or civil aviation. Based on this assumption, the individual safety risk in terms of the acceptable number of deaths per time corresponding to the actual safety performance when travelling by rail or air (3 × 10^−8^/ h) is converted into the average probability of a dangerous car failure per 1 h, i.e. 3 × 10^−6^/ h. Common Safety Method—Design Targets are then used as risk acceptance principles and risk acceptance criteria to harmonise the safety target for SDCs. The resulting harmonised high-level design safety target for self-driving cars PF_SDC_ is 1 × 10^−7^/ h. An example of the allocation of PF_SDC_ to individual safety functions of SDC, including GNSS-based positioning, is illustrated using the fault tree.

Automated car driving (AD) can prevent many commonly occurring car accidents, because it is not tired, sleepy, distracted, tipsy, etc. However, there may also happen so-called edge cases (rare dangerous events) that AD cannot handle. This can be caused, e.g., by sudden changes in the operating environment, human misuse of the AD (incorrect takeover of driving), limited sensor performance under fault-free conditions, etc. For these and other reasons, the safety of the current automated driving systems does not reach the level of safety that a human driver can provide^26^. And yet, as the recent public opinion poll mentioned above shows, SDCs should be as safe as travelling by train or plane. This corresponds to the design target proposed in this paper for SDCs with an average failure probability of 1 × 10^−7^/h. It is therefore clear that this requirement will not be easy to meet. On the other hand, however, if SDCs fail to meet the required level of safety, public will not trust and use them.

The proposed approach backed by the experience and acceptance of railways stakeholders could help the automotive sector for safety demonstration, certification, and approval of automated driving, and also for the process of standardising high-level safety requirements and liabilities for interoperable self-driving vehicles and smart roads.

## Abbreviations

ACSF: Automatically commanded steering function
ALARP: As low as reasonably practicable
CoP: Codes of practice
CSM: Common safety method
CSM-DT: Common safety method design targets
CSM-RA: Common safety method for risk evaluation and assessment
ERTMS: European railway traffic management system
FF: Failure frequency
GAME: Globalement au moins équivalent
GNSS: Global navigation satellite system
HR: Hazard rate
IRF: Individual risk of fatality
MEM: Minimum endogenous mortality
PF: Probability of dangerous failure
RAC: Risk acceptance criteria
RAP: Risk acceptance principles
SAE: Society of automotive engineers
SDC: Self-driving car
SIL: Safety integrity level
TFR: Traffic fatality rate
THR: Tolerable hazard rate
TIR: Target individual risk of fatality
TS: Technical systems
TSI: Technical specification for interoperability
